# Supplementary figures and images for: Computational analysis on two putative mitochondrial protein-coding genes from the Emydura subglobosa genome: A functional annotation approach
Source: PLoS One. 2022 Aug 18;17(8):e0268031. doi: 10.1371/journal.pone.0268031 (PMC9387794; doi:10.1371/journal.pone.0268031)

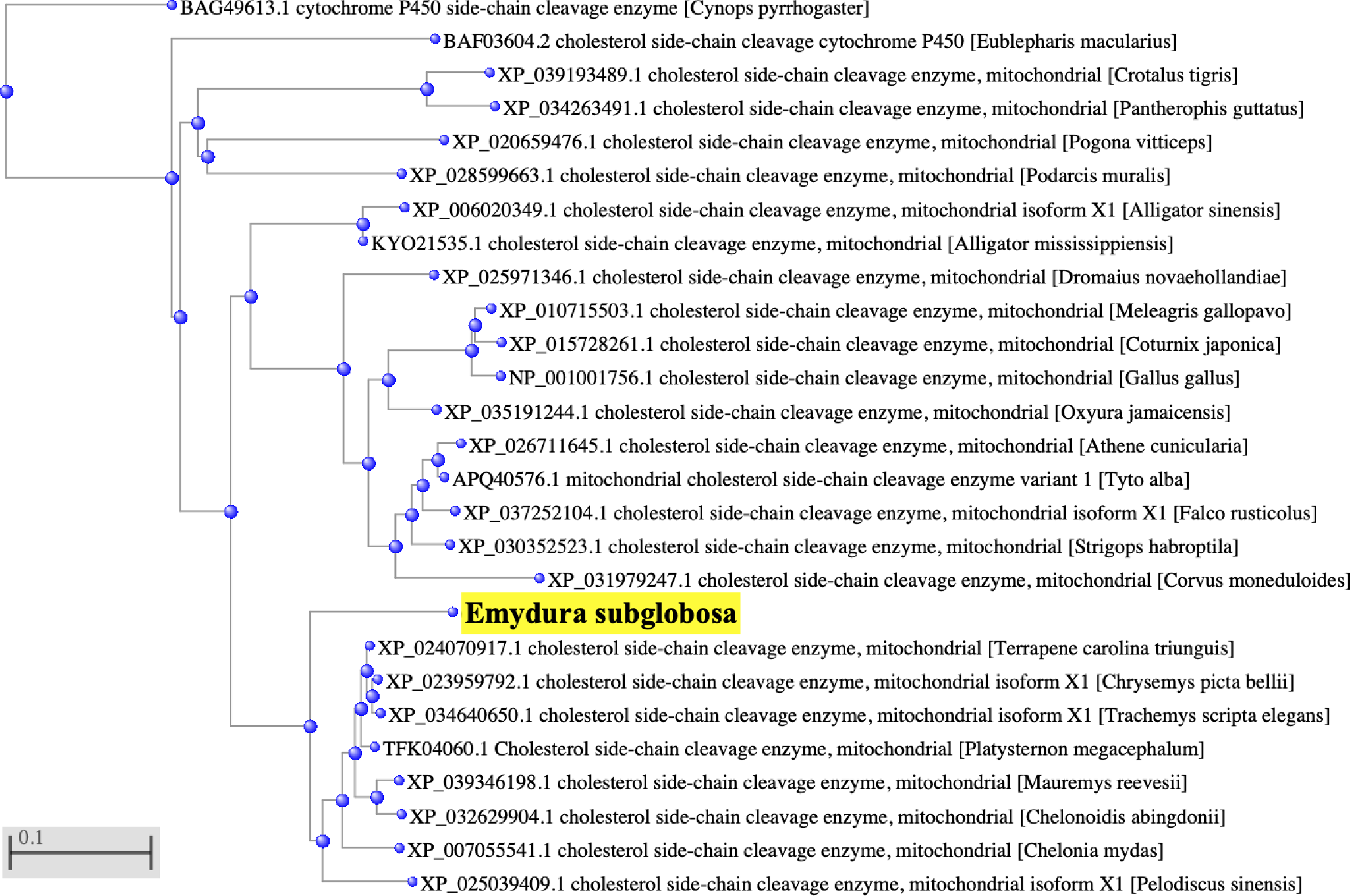

Supplement: S1 Fig — (TIF) [file pone.0268031.s001.tif]

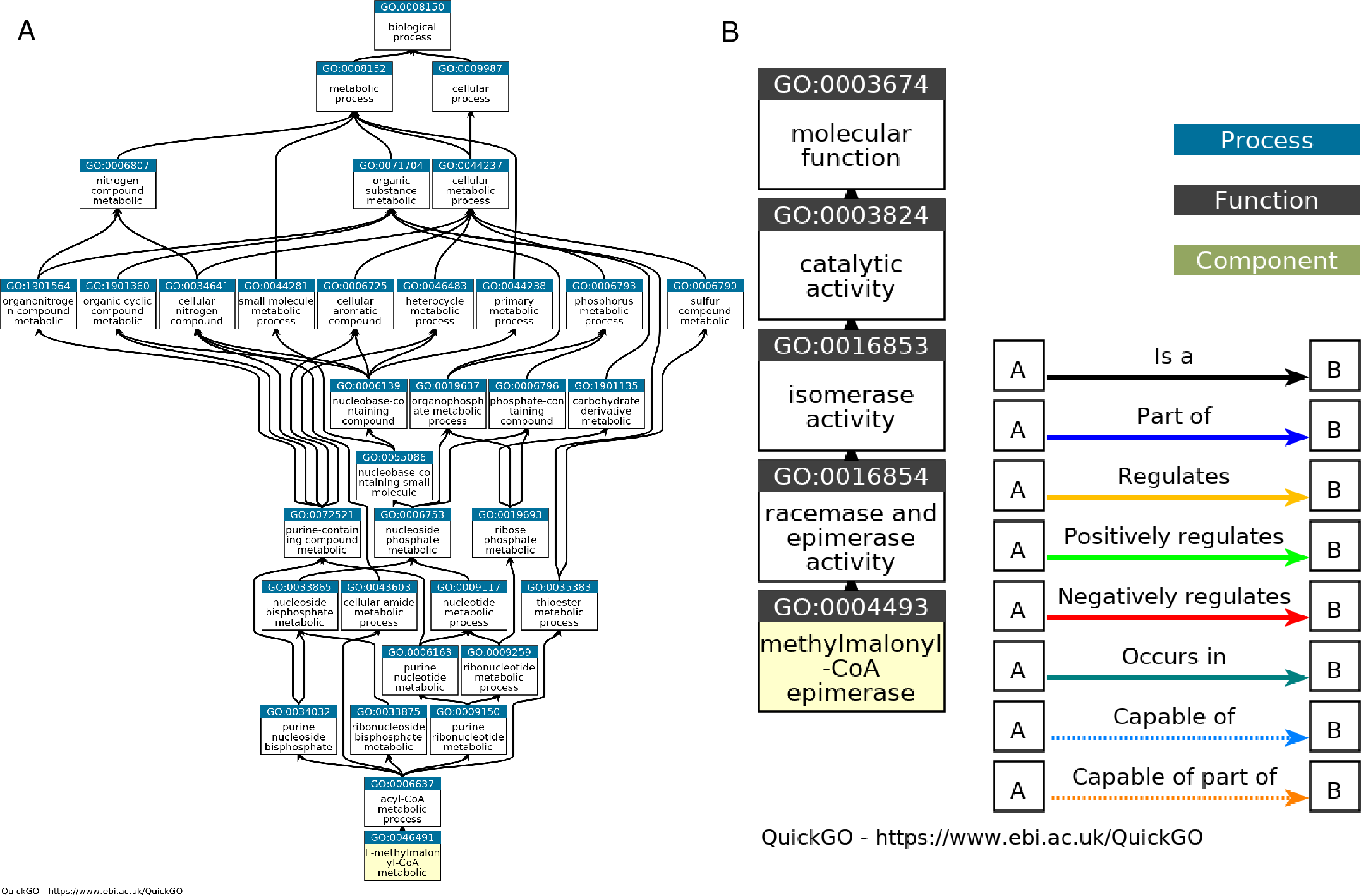

Supplement: S2 Fig — (A) Biological process L-methylmalonyl-CoA metabolic (GO:0046491). (B) Molecular function methylmalonyl-CoA epimerase (GO:0004493). (TIF) [file pone.0268031.s002.tif]

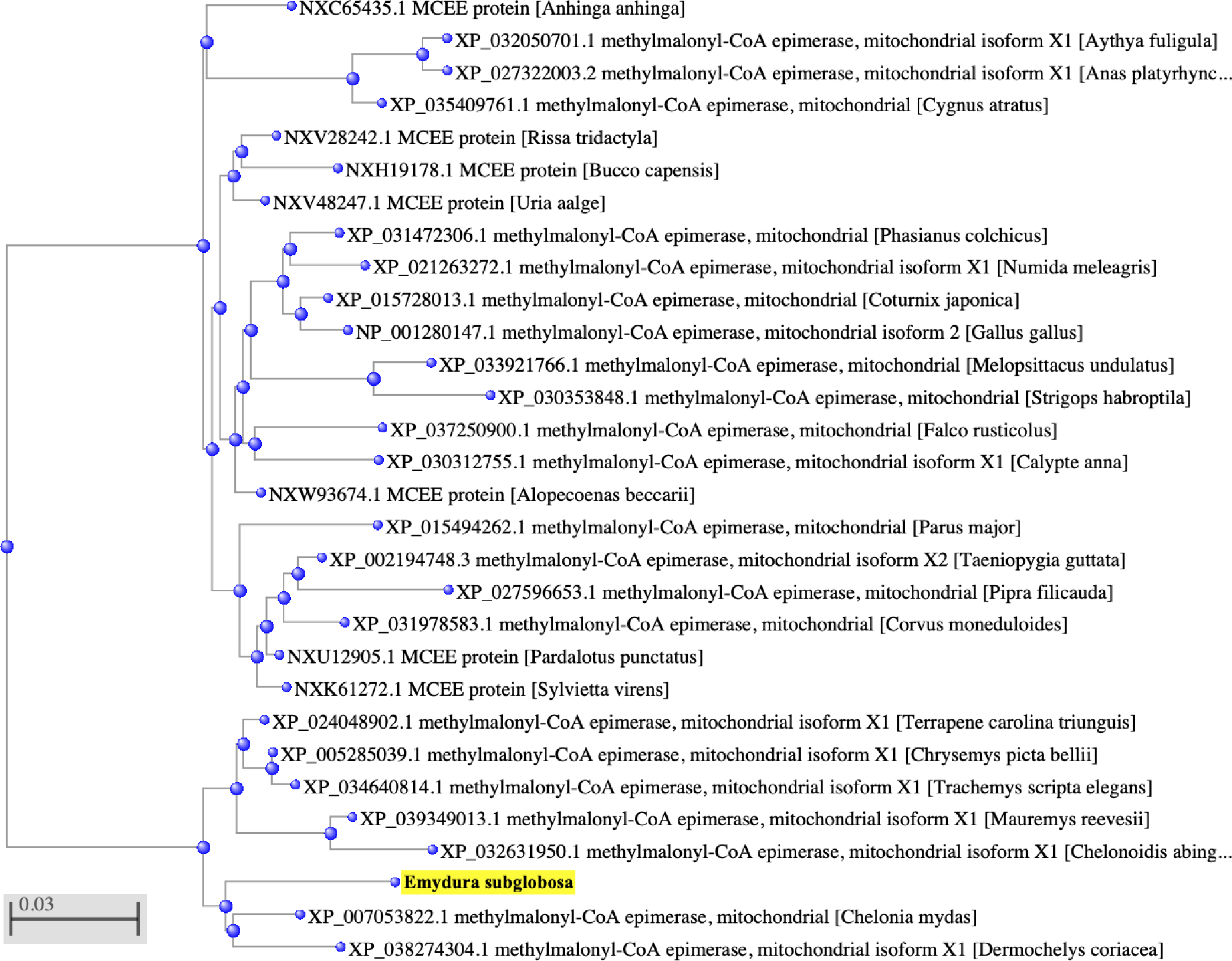

Supplement: S3 Fig — (TIF) [file pone.0268031.s003.tif]
