# Supplementary material for: Computational analysis on two putative mitochondrial protein-coding genes from the Emydura subglobosa genome: A functional annotation approach
Source: PLoS One. 2022 Aug 18;17(8):e0268031. doi: 10.1371/journal.pone.0268031 (PMC9387794; doi:10.1371/journal.pone.0268031)
Supplement: S1 Table — (PDF) [file pone.0268031.s004.pdf]

**Putative Protein**	**Amino Acid Sequence**
Mitochondrial Cholesterol Side-Chain Cleavage Enzyme (CYP11A1)	MLARGGLRLPALAHPSSLWTLSPTEDTQAPGYRRAYMASGEVYPPSLQGRLARPFSELPG<br>NWKTGWLNLYHFWQEGGFHNVHNIMVHKFQKFGPIYREKLGLYESVNIINPEDAATLFKS<br>EGTYPERFMVPPWVAYRDFRNKPYGVLLKKGEAWRSDRLVLNKEALSLQVIDCFVPLLNE<br>VGEDFVKRVRVQIEKSGRGRWTANLTNELFRFALESVCNVLYGARLGLLQDFIDPEAQKF<br>IDAVTLMFHTTSPMLYIPPSLLRRISSKTWRDHVQAWDVIFMHADKCIQTIYRELRLNRK<br>STKEYTGILSSLLVQDKLHIDDIKASVTEMMAGGVDTTSMTLQWAMFELARSPAVQEQLR<br>AEIFAARRAAQGDVLKMLKSIQLLKAAIKETLRIFHTRHWRNFQKIAPALIDSNVVTLVQ<br>VGIYALGRDPHFFPKPELFDPQRWLKTDSTYFKGLGFGFGPRQCLGRRIAELEMQLFLIH<br>MLENFKIETKRGVDIGTKFDLILIPDKPIHLTLRALDSLP
Mitochondrial Methylmalonyl-CoA Epimerase (MCEE)	(before gene editing)<br>MAAACMGKAAAAGLLTRLQTTAATVRTLSMSHSLTQKVPCSLWKLGRLNHIAIAVPDLEK<br>AQSFYKSVLGAQVDDIKAAMAELKEKKIRILSEEAKIGAHGKPVIFLHPKDCDGVLIELE<br>QA
	(after gene editing)<br>MAACMGKAAAGLLTRLQTTAATVRTLSMSHSLTQKVPCSLWKLGRLNHIAIAVPDLEKAQ<br>SFYKSVLGAQVSETVPLPEHGVYTIFVELGNTKLELLYPLGEKSPIAGFLQKNKAGGMHH<br>ICIEVDDIKAAMAELKEKKIRILSEEAKIGAHGKPVIFLHPKDCDGVLIELEQA
