# Supplementary material for: Computational analysis on two putative mitochondrial protein-coding genes from the Emydura subglobosa genome: A functional annotation approach
Source: PLoS One. 2022 Aug 18;17(8):e0268031. doi: 10.1371/journal.pone.0268031 (PMC9387794; doi:10.1371/journal.pone.0268031)
Supplement: Attachment — Submitted filename: Rebuttal Letter PLOS.pdf [file pone.0268031.s005.pdf]

Megan Yu
Department of Molecular, Cell & Developmental Biology
University of California, Los Angeles
Los Angeles, CA 90095

April 5, 2022

Rakesh Kumar Verma, Ph.D.
Academic Editor
PLOS ONE

Scott A. Thomson
Reviewer
PLOS ONE

Dear Dr. Rakesh Kumar Verma and Dr. Scott A. Thomson,

Thank you for your time and feedback in reviewing my original research article entitled "Computational analysis on two putative mitochondrial protein-coding genes from the *Emydura subglobosa* genome: A functional annotation approach." I have addressed the reviewer's comments and clarified specific phrases that may have been confusing to the reader:

*1. **Line 42**: Compared to other eukaryotic species, turtles have minimal diversity (360 living species) and over 50% are threatened with extinction [2].*

I find this sentence a little awkward and open to interpretation. "other eukaryotic species' groups" of something similar may be better as it clarifies we you are talking about Orders in this case. Also historically there have been in the past many more species than are alive today there are many hundreds of fossil species, probably comparable with other reptilian orders.

I agree that talking about individual species may be ambiguous, as I am focusing on Orders in this sentence. I have changed the wording to "other eukaryotic species' groups" to clarify this.

*2. In the **materials and methods** section the writing is as if there were multiple people involved here but there is a sole author. This may read better under the circumstances with some better use of third person writing rather than first person. This is just a suggestion the materials and methods is otherwise thorough.*

This point is valid. I am used to writing in first-person, especially since most papers I have worked on have multiple authors. I have removed the first-person language and changed to third person instead.

*3. **Line 317**: when you refer to "other turtle species" are you referring to previously studied taxa you mentioned in the intro? Such as C. n. abingdoni, G. gigantea and C. mydas? Maybe that should be clarified as E. subglobosa is to my knowledge the first Pleurodiran turtle to be studied this way, all others being Cryptodires, as such E. subglobosa being an outgroup or sister to all other studied turtles would be an expected result as at least 165 million years separates them. **Addit**. Ok I think I get it. It's the species listed in your tables? you may want to make this clear somewhere early in the manuscript, ie material examined so the reader knows what your comparative material is.*

I have clarified this point specifically in the Materials and Methods section Lines 289-303. For "other turtle species," I am referring to the species in the phylogenetic tree, which I obtained from the BLAST results. I expanded out the organism sets in the phylogenetic tree to contain species with lower percent

identities in the BLAST results. I clarified “other turtle species” in Line 375-376 to “other turtle species in the phylogenetic tree.” Readers can refer to the Materials and Methods for how this tree was generated.

I have also reviewed the journal guidelines and ensured that my manuscript meets PLOS ONE’s style requirements. I confirm that I am affiliated with the University of California, Los Angeles (UCLA) Department of Molecular, Cell & Developmental Biology. I did not update any figures for this revision, and all figures were uploaded to PACE. All references are complete and correct.

Please address correspondence to me via email at yumr247@g.ucla.edu.

Thank you very much for your time and consideration of this manuscript. I look forward to hearing back from you and *PLOS ONE*.

Sincerely,

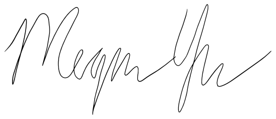

Megan Yu
Corresponding Author
